# Two‐Arm Crossover Randomized Controlled Trial Versus Meta‐Analysis of N‐of‐1 Studies: Comparison of Statistical Efficiency in Determining an Intervention Effect

**DOI:** 10.1002/bimj.70045

**Published:** 2025-03-12

**Authors:** Anna Eleonora Carrozzo, Georg Zimmermann, Arne C. Bathke, Daniel Neunhaeuserer, Josef Niebauer, Stefan T. Kulnik

**Affiliations:** ^1^ Ludwig Boltzmann Institute for Digital Health and Prevention Salzburg Austria; ^2^ Faculty of Digital and Analytical Sciences Paris‐Lodron University Salzburg Salzburg Austria; ^3^ Salzburg Research Forschungsgesellschaft m.b.H Salzburg Austria; ^4^ Intelligent Data Analytics (IDA) Lab Salzburg Department of Artificial Intelligence and Human Interface (AIHI) Faculty of Digital and Analytical Sciences Paris‐Lodron University Salzburg Salzburg Austria; ^5^ Team Biostatistics and Big Medical Data IDA Lab Salzburg Paracelsus Medical University Salzburg Austria; ^6^ Research Programme Biomedical Data Science Paracelsus Medical University Salzburg Austria; ^7^ Sport and Exercise Medicine Division Department of Medicine University of Padova Padua Italy; ^8^ Institute for Molecular Sports and Rehabilitation, Paracelsus Medical University Salzburg Austria; ^9^ University Institute of Sports Medicine, Prevention, Paracelsus Medical University Salzburg Austria

**Keywords:** comparative effectiveness, crossover Design, meta‐analysis, N‐of‐1 trials, two‐arm randomized controlled trial

## Abstract

N‐of‐1 trials are currently receiving broader attention in healthcare research when assessing the effectiveness of interventions. In contrast to the most commonly applied two‐arm randomized controlled trial (RCT), in an N‐of‐1 design, the individual acts as their own control condition in the sense of a multiple crossover trial. N‐of‐1 trials can lead to a higher quality of patient by examining the effectiveness of an intervention at an individual level. Moreover, when a series of N‐of‐1 trials are properly aggregated, it becomes possible to detect an intervention effect at a population level. This work investigates whether a meta‐analysis of summary data of a series of N‐of‐1 trials allows us to detect a statistically significant intervention effect with fewer participants than in a traditional, prospectively powered two‐arm RCT and crossover design when evaluating a digital health intervention in cardiovascular care. After introducing these different analysis approaches, we compared the empirical properties in a simulation study both under the null hypothesis and with respect to power with different between‐subject heterogeneity settings and in the presence of a carry‐over effect. We further investigate the performance of a sequential aggregation procedure. In terms of simulated power, the threshold of 80% was achieved earlier for the aggregating procedure, requiring fewer participants.

## 1 Introduction

The advent of “personalized medicine” has led to an increased interest in “single‐case” or “single‐subject” approaches (Barlow and Hersen 1984). In such approaches, also known as N‐of‐1 trials, a single subject undergoes a sequence of interventions (typically, an experimental vs. a control condition) in a random order to identify the best personalized strategy (Guyatt et al. 1986). For this reason, it fits well in situations where the treatment can be implemented and withdrawn with minimal concern about a carry‐over effect, and the treatment results in concurrent symptomatic improvement, rather than a permanent change in disease status. Indeed, since suspending the treatment is supposed to bring the subject back to the baseline, ethical issues might arise. In evaluating digital health interventions, this aspect does not necessarily present a problem. Consider, for example, a digital intervention with the aim to motivate people to be physically active such as a digital planning calendar for physical activity that allows prompt notifications when an activity is planned. Withdrawing the intervention (prompt notifications) appears reasonable and does not present a significant health risk to the participant. Furthermore, a carry‐over effect of such an intervention may be considered negligible in a short period. Note that throughout the paper, the terms “treatment,” “intervention,” and “experimental condition” are used synonymously.

Subjects can cross between experimental and control conditions many times (blocks or cycles). More block repetitions ideally correspond to higher information about the within‐subject treatment effect. Thus, these multi‐crossover N‐of‐1 trials can potentially provide evidence‐based information for individual patients, resulting in higher quality of patient care (Mahon et al. 1996; Duan et al. 2013).

There is still a lack of consensus about the optimal approach to analyzing N‐of‐1 data (Manolov and Onghena 2024). Gabler et al. (2011) reported a systematic review of methodologies used to analyze N‐of‐1 trials. The majority revolves around visual/graphic representations with no statistical comparisons but also parametric tests such as independent *t*‐tests, paired *t*‐tests, and analysis of variance are used when data follow a normal distribution. Bayesian approaches have also been proposed recently (Liao et al. 2023). Furthermore, results of N‐of‐1 trials conducted on a coherent population can be combined to estimate a population‐level treatment effect (D. R. Zucker et al. 2010). A series of N‐of‐trials conducted under a similar protocol is essentially a multi‐crossover design. Approaches for analyzing data from a series of N‐of‐1 thus also fall within the realm of crossover trials. However, some methods for multi‐crossover studies are relatively complex and revolve around regression models with mixed effects (B. Jones and Kenward 2003). Approaches for combining individual results from a series of N‐of‐1 trials further include meta‐analysis of summary data, multilevel models, and Bayesian hierarchical models (D Zucker et al. 1997; Higgins et al. 2001; Huber et al. 2007; A. P. Jones et al. 2009; Yang et al. 2021).

This work is inspired by a real‐world scenario raising the question about the most appropriate study design to evaluate a digital health intervention in cardiovascular care. In particular, we aim to assess if an aggregating approach of N‐of‐1 trials might lead to the same conclusions as a randomized controlled trial (RCT) or a crossover trial more efficiently, that is, with fewer participants. In this work, we consider the aggregating approach as a meta‐analysis of summary data from a series of N‐of‐1 trials. Furthermore, we aim to investigate the performance of a sequential aggregation procedure, that is, as each new participant enters the study.

The paper is organized as follows: Section 2 introduces the motivational example which inspired this work. In Section 3. a description of the procedures and methods is given. Section 4 introduces some analytical considerations about the sample size requirement of each study design considered. Section 5 presents a simulation study to further compare the performance of the considered methods. Section 6 discusses the findings and limitations and possible extensions.

## 2 Motivational Example

After a cardiovascular disease (CVD) event, exercise‐based cardiac rehabilitation constitutes an important care pathway for the secondary prevention of CVD. Cardiac rehabilitation programs aim to support patients in implementing a lifelong healthy lifestyle (Kotseva et al. 2019). Indeed, as a general recommendation, patients should carry out at least 150 min per week of moderate or 75 min per week of vigorous physical activity. The cardiac rehabilitation pathway consists of different phases (typically three) (Ambrosetti et al. 2021). Phase I describes the period of acute hospital admission where patients recover from an acute event such as a heart attack or cardiac surgery. Phase II, typically lasting 3–6 weeks, describes a structured program, delivered in an inpatient or outpatient setting, with the aim to promote independence and lifestyle changes to prepare patients to return to their lives at home. Phase III describes the lifelong self‐management of a heart‐healthy lifestyle. Patients receive encouragement towards maintaining an active lifestyle and continue the exercise. However, successful completion of a phase II cardiac rehabilitation program does not guarantee that a long‐term physical activity habit is established. The aktivplan digital health intervention (Marcos et al. 2024) was developed to support patients in establishing regular physical activity habits following the completion of a phase II rehabilitation program in the Austrian healthcare context. The intervention is a digital planning calendar for regular physical activity implemented into an app. Patients and healthcare professionals agree on a physical activity plan. Reminder notifications on the days with a planned physical activity session can be sent automatically by the app. In order to assess whether this intervention leads to more physical activity with respect to usual care, a 12‐month trial is being planned. One outcome that can be considered to reflect the effectiveness of the intervention is the daily minutes of moderate to vigorous physical activity (MVPA) assessed by accelerometry. The RCT is the gold standard design for evaluating new interventions. However, an RCT could be very resource‐intensive in terms of sample size requirements. In an RCT, study participants are randomized to the intervention group (aktivplan) or control (standard care, i.e., not including any routinely provided digital technologies). The generic formula for the number of participants for each group to test the difference between two independent groups, achieving a prespecified type I, α and type II, β error rate is $n=2\sigma^{2}{\left\{(z_{1-\alpha /2}+z_{1-\beta })/\Delta \right\}}^{2}$. Using data from a completed cardiac rehabilitation trial (Claes et al. 2017, 2020), the standard deviation of MVPA is σ=64.5. We consider Δ=10 min/day as a minimal clinically meaningful change to be detected. With α=0.05 and β=0.2, the number of participants necessary for each group should be 652. May other approaches be more efficient in terms of sample size requirements in detecting the intervention effect?

A crossover design can also be considered in the following setting. Subjects are randomized at training begin to a sequence of interventions (aktivplan—standard of care or standard of care—aktivplan) and then perform two 6‐month periods of training accordingly. During the standard of care period, aktivplan is deactivated. Another setting can be that subjects are randomized to a sequence of interventions (aktivplan—standard of care or standard care—aktivplan), then perform two 2‐month periods of training accordingly and repeat three cycles. In what follows, we aim to compare these three approaches through a simulation study. The next section introduces the model assumptions and the methodological procedures compared.

## 3 Methods

Let us assume that we want to compare two conditions, one control (A) and one experimental (B), over *J* cycles. Consider the general model:

(1) $$Y_{ijk}=\mu +\gamma_{i}+\delta Z_{ijk}+\varepsilon_{ijk},$$

where $Y_{ijk}$ and $Z_{ijk}$ are, respectively, the observed value of the outcome of interest and the treatment indicator for subject i, i=1,…,N, at cycle j,j=1,…,J and period k,k={1,2}. μ is the general population mean, and $\gamma_{i}∼N\left(0,\tau^{2}\right)$ is the random effect for patient *i*, and it is assumed to have a common variance across all subjects. δ represents the treatment effect and depending on i,j,k, δ can be $\delta_{A}$ or $\delta_{B}$ that represent the effects produced by the two treatments A and B, respectively. Without loss of generality, let us assume that $\delta_{A}=0$ and $\delta_{B}=\delta \geq 0$. $\varepsilon_{ijk}∼N\left(0,\sigma_{i}^{2}\right)$ are random error terms that are assumed to be independent from occasion to occasion. $\tau^{2}$ describes the variance between subjects and represents the heterogeneity, the greater is this variance the greater is the variation of the outcome from subject to subject. $\sigma_{i}^{2}$ represents the within‐subject variance, that is, the measurement variance expected to occur if the measurement procedure would be repeated a large number of times in the same patient under identical conditions and can be assumed known and equal to the estimate of each single‐subject study. $\tau^{2},$ on the contrary, is not known and may be estimated using different methods (DerSimonian and Laird 1986; Whitehead and Whitehead 1991; Hardy and Thompson 1996; Partlett and Riley 2017). In what follows we formalize the three trial designs and statistical methods that can be adopted to compare the two conditions.

### 3.1 Parallel RCT

This setting represents the traditional RCT. Each subject is randomized to receive one of the two treatments A or B. In this situation, we have only one cycle (J=1) with one period (k=1) for each subject. The subjects perform a training period with the corresponding treatment and the outcome Y thought to be affected by the treatment is measured. An unpaired *t*‐test is performed between the two groups receiving different conditions to test the set of hypotheses, $H_{0}:Y_{A}=Y_{B}$ against $H_{1}:Y_{A}\neq Y_{B}$.

### 3.2 Two‐Period Crossover Design

In this setting, we have one cycle (J=1) with two periods (k={1,2}). Each subject is randomized to a treatment sequence (A–B or B–A) and undergoes two subsequent study periods accordingly. To test the treatment effect, we consider the within‐subject difference in outcome between the two study periods $D_{i}\left(AB\right)=Y_{i1k=2}-Y_{i1k=1}$ for iin{AB} and $D_{i}\left(BA\right)=Y_{i1k=2}-Y_{i1k=1}$ for iin{BA} and then perform an unpaired *t*‐test between the two sequence groups (Wellek and Blettner 2012).

### 3.3 Meta‐Analysis of Summary Data From N‐of‐1 Trials

In this setting, we have J≥2 cycles per subject. Thus, each subject is randomized to a treatment sequence (A–B or B–A) as the trial begins and undergoes J cycles of the corresponding sequence. We consider the within‐subject difference in each cycle $D_{ij}=Y_{ijk=2}-Y_{ijk=1}$ for iin{AB} and $D_{ij}=Y_{ijk=1}-Y_{ijk=2}$ for iin{BA}. Then we combine these differences within the subject by ${\hat{\theta }}_{i}=\frac{1}{J}\sum_{j=1}^{J}D_{ij}$. The mean differences of each single study will be aggregated as if they were single means of studies in a meta‐analysis. Denoting an estimate of the mean from subject *i* by ${\hat{\theta }}_{i}$ and the reciprocal of its estimated variance by $w_{i}$, a traditional estimate of the overall mean θ is obtained using the weighted average $\hat{\theta }=\left(\sum w_{i}{\hat{\theta }}_{i}\right)/\left(\sum w_{i}\right)$, where $w_{i}=1/\left(\sigma_{i}^{2}+\tau^{2}\right)$. A confidence interval is computed as $\;\hat{\theta }\pm z_{1-\alpha /2}\sqrt{V(\hat{\theta })}$, where $\sqrt{V(\hat{\theta })}=1/\sum w_{i}$.

## 4 Sample Size: Analytical Considerations

To assess which design is more efficient in terms of sample size requirement to achieve adequate statistical power, let us make some considerations about the different factors and parameters characterizing each method. As mentioned, the generic formula for the number of participants for each group in a parallel RCT, achieving a prespecified type I, α and type II, the β error rate is $n_{RCT}=2\sigma^{2}{\left\{(z_{1-\alpha 2}+z_{1-\beta })/\Delta \right\}}^{2}$. The sample size formula for the parametric analysis of a crossover design under the usual statistical model assumptions will be $n_{co}=\sigma_{e}^{2}{\left\{\left(z_{1-\alpha }+z_{1-\beta }\right)/\left(\Delta \right)\right\}}^{2}$ (Grizzle 1965), where $\sigma_{e}^{2}$ is the within‐subject variance. When conducting multiple N‐of‐1 trials and aggregating the results through meta‐analysis, the total sample size across individuals is a combination of the within‐subject comparisons from each individual trial, accounting for between‐subject heterogeneity ($\tau^{2}$). The formula for the effective sample size would be $N_{meta}=\left(\sigma_{e}^{2}+\tau^{2}\right){\left\{(z_{1-\alpha /2}+z_{1-\beta })/\Delta \right\}}^{2}$.

Thus, for the parallel RCT, the total sample size depends on the between‐subject variance $\sigma^{2}$, which includes variability across individuals who receive only one treatment or control. On the contrary, since the crossover design is based on within‐subject comparisons, the total sample size depends on the within‐subject variability $\sigma_{e}^{2}$ that is typically smaller than $\sigma^{2}$. This makes crossover designs in general more efficient than the parallel RCTs. The aggregation through meta‐analysis on N‐of‐1 trials is influenced by both within‐subject variance $\sigma_{e}^{2}$ and between‐subject heterogeneity $\tau^{2}$, both of which are generally smaller than the full between‐group variability in the parallel design. The sample size requirement for a meta‐analysis of N‐of‐1 trials is generally comparable to that of a two‐period crossover design, but the presence of $\tau^{2}$ can lead to a slightly higher sample size.

Thus, in general, both the crossover design and meta‐analysis of N‐of‐1 trials tend to be more efficient than a parallel two‐arm RCT because they exploit within‐subject comparisons reducing variance. However, analytical methods, while useful for generalizing broad trends, quickly become intractable unless one relies on simplifying assumptions (e.g., homoscedasticity or negligible carry‐over effects) that may not realistically reflect the data. Indeed, the crossover design eliminates between‐subject variability $\left(\tau^{2}\right)$ in the estimation of treatment effects, which may underestimate the true impact of heteroscedasticity in certain scenarios. Furthermore, the presence of treatment carry‐over effects, which may not be fully captured in analytical comparisons, could diminish the efficiency advantage of a crossover design.

To address these complexities, Monte Carlo simulation studies can provide a valuable complement to analytical methods. Simulations allow the evaluation of various scenarios, such as different levels of within‐subject variance, heterogeneity, and residual carry‐over effect, or considering distributions outside the analytically tractable classical distribution families. By incorporating these options, simulation studies offer greater depth, flexibility, and practical insight into the relative performance of competing methods under realistic conditions.

## 5 Simulation Study

A simulation study was performed to empirically compare the performances of the three methods described in Section 3 in identifying a treatment effect δ in a control versus treatment comparison, in terms of power and sample size requirement. We simulated data from the model (Barlow and Hersen 1984) with μ=0 and J=3 cycles for the N‐of‐1 trials. We considered different situations for the random effect variance, τ=0,0.1,0.5 for simulating different between‐subject heterogeneity scenarios. The standard deviation for the error term within each study was randomly generated and uniformly distributed between 0.05 and 1 for each simulation scenario. The first cycle's observations with the two sequences (A–B, B–A) were used for the crossover trials and the first period only of the first cycle, for each treatment assignment for the RCTs. We considered three settings of heterogeneity: (a) homogeneity (τ=0), (b) weak heterogeneity (τ=0.1), and (c) strong heterogeneity (τ=0.5). The following approach was applied to investigate the performance of the three methods in detecting the treatment effect. For a fixed treatment effect, we empirically determine the power of each procedure for increasing sample size. Then for a fixed sample size, we compared the methods both when no treatment effect was present and for increasing treatment effect. We further investigated the effect of a carry‐over effect by increasing the outcomes of subjects who received condition A (control) after condition B (experimental). No carry‐over effect is present in RCTs. Thus, to simulate a carry‐over effect in the crossover trials, we added 10% of the treatment effect (B) to the outcome of the second period of the subjects in the B–A sequence group, and also to that of the first period in the second and third cycle of the subjects in the A–B sequence group, in the N‐of‐1 trials. For each simulation setting, 5000 simulation runs were performed and we computed the rejection rate for each of the three methods. The analysis was performed using the statistical software R, and in particular the “metamean” function from the package “meta” (Balduzzi et al. 2019) for the meta‐analysis of the series of N‐of‐1 trials. We used the Kenward–Roger method as the estimator of the between‐study variance $\tau^{2}$ (Partlett and Riley 2017). This method is based on an adjusted variance estimate for the random effects estimate. This part of the simulation study aimed to compare the performances of the three study setups to give an indication of which one could be more appropriate when planning a trial.

Furthermore, we separately investigated the behavior of the aggregated N‐of‐1 procedure sequentially through a cumulative meta‐analysis. Due to the computational complexity, we only performed 100 simulations of n=100 N‐of‐1 trials for each treatment sequence, under setting c (strong between‐subject heterogeneity), and with a carry‐over effect. For each simulated series of 2n N‐of‐1 trials, we randomly permute the order to mimic random allocation to the treatment sequence of the subjects that sequentially enter the study. Then the cumulative meta‐analysis was conducted, adjusted by a Bonferroni correction at each step to take into account possible multiple testing issues. Finally, following a sequential approach, the “rejection” or “acceptance” at each step of aggregation was defined as follows: if and only if at least one among the current or the previous steps was rejected, then the current step was considered rejected. This part of the simulation study instead wanted to explore the number of aggregations of single N‐of‐1 studies, possibly coming from different studies with similar protocols but performed at different times, which are necessary to evaluate the difference between two interventions at a population level.

## 6 Results

**FIGURE 1:**
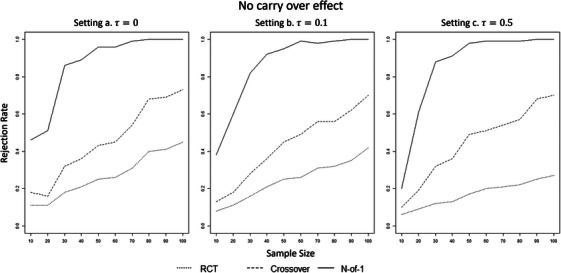
Rejection rates of the three methods for δ=0.15 for increasing sample sizes, with no carry‐over effect, for setting (a) homogeneity (τ=0), (b) weak heterogeneity τ=0.1, and (c). strong heterogeneity τ=0.5.

**FIGURE 2:**
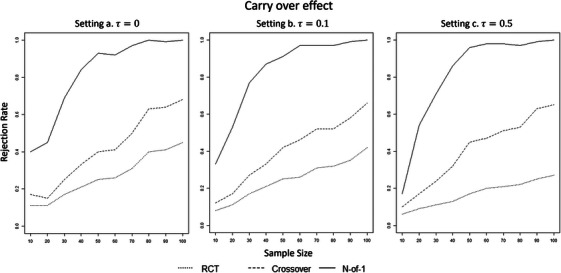
Rejection rates of the three methods for δ=0.15 for increasing sample sizes, with carry‐over effect, for setting (a) homogeneity (τ=0), (b) weak heterogeneity τ=0.1, and (c) strong heterogeneity τ=0.5.

First, we investigated the empirical power (rejection rate [RR]) achieved by each procedure to detect a fixed treatment effect δ=0.15 for different sample sizes (n=10,20,30,…,100). The sample size n is the number of subjects in each treatment group or sequence group. Thus, n=10 means that 10 subjects received condition A and 10 condition B in the RCT, whereas 10 received conditions in the order A–B and 10 in the order B–A in the crossover and N‐of‐1. Figures 1 and 2 show the results for the different heterogeneity settings without and with carry‐over effects, respectively. The aggregated N‐of‐1 overperformed both RCT and crossover designs in all situations and settings. In particular, the aggregated N‐of‐1 achieved the empirical power of 0.80 around n=30 ($RR_{a}=0.86,\;RR_{b}=0.82,\;RR_{c}=0.88$) when no carry‐over effect was present and with n=40 with a carry‐over effect ($RR_{a}=0.84,\;RR_{b}=0.87,\;RR_{c}=0.86$). The procedure did not seem to be affected by increasing heterogeneity. The other two methods did not achieve the rejection rate of 0.80 for any of the sample sizes considered. Nevertheless, the crossover design performed better than the RCT. Furthermore, the RCT appears to be affected more by the heterogeneity. For n=100, the crossover design obtained an empirical power of $RR_{a}=0.73,\;RR_{b}=0.70,\;RR_{c}=0.70$ with no carry‐over effect and of $RR_{a}=0.68,\;RR_{b}=0.66,RR_{c}=0.65$ with carry‐over effect, whereas the RCT of $RR_{a}=0.45,\;RR_{b}=0.42,RR_{c}=0.27$ (no carry‐over effect for RCTs). The crossover design exceeded the power of 0.8 for n=150 ($RR_{a}=0.88,RR_{b}=0.88,\;RR_{c}=0.88$ with no carry‐over effect, and $RR_{a}=0.85,\;RR_{b}=0.85,RR_{c}=0.85$ with carry‐over effect). The RCT reached the power of 0.80 for $n=300\;(RR_{a}=0.88,\;RR_{b}=0.86)$ for setting with homogeneity and low heterogeneity and for n=420 ($RR_{c}=0.81$) for a setting with strong heterogeneity.

**FIGURE 3:**
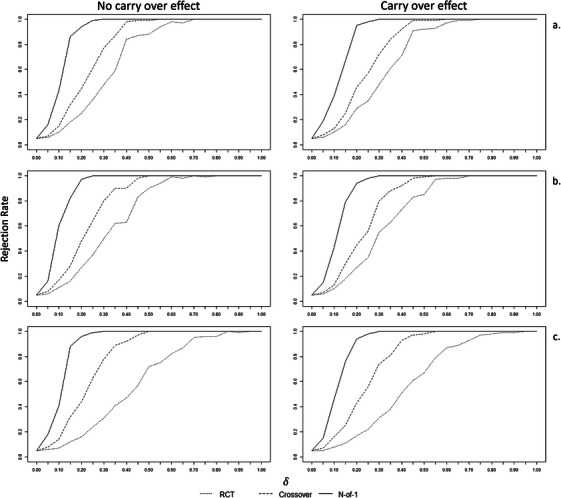
Rejection rates of the three methods for fixed sample size *n* = 30 with increasing true treatment effect δ, without and with carry over effect, for setting (a) homogeneity (τ=0), (b) weak heterogeneity τ=0.1, and (c) strong heterogeneity τ=0.5.

Second, for fixed sample size n=30 we investigated the behavior of the three methods as the true treatment effect increases δ=0,0.05,0.10,…1. For δ=0, that is, under the null hypothesis of no treatment effect, all the methods matched the empirical type I error rate of 0.05 in all settings and situations. Figure 3 shows that the aggregated N‐of‐1 approach outperformed both crossover and RCT designs as δ increased. The power of 0.80 is reached for the N‐of‐1 approach for δ=0.15 and was very close also with a carry‐over effect ($RR_{a}=0.67,\;RR_{b}=0.79,\;RR_{c}=0.76$). It exceeded the power of 0.80 for δ=0.20 for all settings. The crossover design achieved the power of 0.80 for δ=0.35 for all settings, whereas the RCT for δ=0.40 for homogeneity, for δ=0.45 for weak heterogeneity, and for δ=0.60 for strong heterogeneity.

**FIGURE 4:**
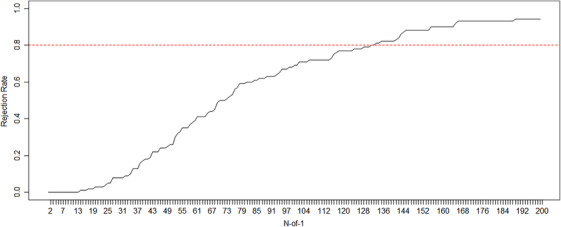
Rejection rate of a cumulative meta‐analysis with a Bonferroni correction to detect a true treatment effect δ=0.15, τ=0.5, and with carry‐over effect.

Finally, we examined the behavior of the meta‐analysis of N‐of‐1 trials sequentially through a cumulative meta‐analysis. Figure 4 shows the rejection rates at each aggregation step over 100 simulations of 2n=200 N‐of‐1 trials, n=100 for each treatment sequence. From a previous simulation study, we found that n=30 for each treatment sequence, was likely a sample size sufficient to detect a treatment effect of δ=0.15 with a power of at least 0.80 within a strong between‐subject heterogeneity setting. However, this cumulative approach requires an adjustment for multiplicity (Bonferroni), making the approach conservative. Indeed, the expected power after 60 sequential aggregations was much lower than 0.80 (0.40). Note also that the 60 sequential aggregations in this cumulative analysis differ from the analysis on 60 N‐of‐1 trials where treatment sequence assignment is balanced (n=30 subjects per treatment sequence). The treatment sequences in the sequential analysis are random at each aggregation step and not necessarily balanced between subjects. In order to detect a treatment effect of δ=0.15 the desired power of 0.80 was achieved after 131 aggregations. This number is more than twice the sample size required by the noncumulative procedure (2n=60), but still lower than that for the RCTs (2n=840) and the crossover design (2n=300).

## 7 Discussion and Conclusions

The most common design in the regulatory approval process to provide a good estimate of a generalizable intervention effect for the given study population is the parallel group RCT. However, this well‐established study design can be affected by a large variability of between‐subject characteristics at the baseline, resulting in large sample size requirements. Conversely, N‐of‐1 trials offer a valuable strategy to provide information for individual patients, enabling unbiased estimation of the intervention effect for single subjects. Moreover, when properly conducted on a sample of subjects from a population of interest, it is possible to estimate a population treatment effect by aggregating results from single N‐of‐1 trials (D. Zucker et al. 1997). In this work, we focused on the meta‐analysis of summary data from single N‐of‐1 trials as an aggregating method of a series of N‐of‐1 studies, and we compared its performance with the parallel and crossover RCT through a simulation study. In our simulation study under the given model, the meta‐analysis of summary data from N‐of‐1 trials overperformed both the crossover design and the parallel two‐arm RCT in terms of efficiency. In particular, to detect the same true treatment effect, it reached the target power of 80% with fewer participants, thus allowing the detection of a relatively small treatment effect with a reasonable sample size. This result highlights the potential of N‐of‐1 trials in leveraging within‐subject comparisons while accommodating heteroscedasticity and between‐subject variability.

Analytical comparisons (Section 4) suggested that the crossover design should generally be more efficient due to its ability to eliminate between‐subject variability. However, simulation results revealed important nuances. The analytical framework may underestimate the impact of heteroscedasticity and not sufficiently account for potential residual carry‐over effects, which could reduce the efficiency of the crossover design. When incorporating these complexities explicitly in a simulation study, the approach based on a meta‐analysis of N‐of‐1 trials exhibited advantages when handling scenarios involving both within‐ and between‐subject variability.

In this work, we considered N‐of‐1 studies with three cycles only. Although more cycle repetitions theoretically correspond to greater information about the within‐subject treatment effect (Senn 2019), the number of cycle repetitions that might correspond to smaller sample size requirements could also result in a longer overall trial time, increased participant burden, and the likelihood of dropouts. These aspects may affect the feasibility in large‐scale of this approach. Another important critical assumption of both N‐of‐1 and crossover designs is the stability of the disease or condition over time. This assumption is essential because changes in the underlying disease trajectory during the study period can confound treatment effects, reducing the validity of within‐subject comparisons. Indeed, this restriction limits the application of N‐of‐1 and crossover designs in clinical research contexts to chronic or stable conditions. The present work follows the motivational example of digital intervention in cardiovascular care, where the time for the trial is fixed (12 months), and we compared different designs and associated analysis strategies. In such a situation, the N‐of‐1 approach does not imply any extension of the trial time. Furthermore, the treatment effect is thought to be relatively stable in time with an immediate effect on the outcome. Regarding our motivational example, a prompt reminder message is likely to have an immediate effect on the intention to do physical activity. A wash‐out period is not likely relevant in this trial setting, instead, it is reasonable to expect only a small immediate residual effect after the intervention period. We hope that the considerations from this comparative study may inform scientists who are planning a trial with similar characteristics.

Furthermore, we examined the situation in which different N‐of‐1 trials are conducted at different times, and we investigated whether it was possible to sequentially aggregate single results to estimate a treatment effect at a population level as each new information is presented. For this purpose, a cumulative meta‐analysis of summary data of single N‐of‐1 trials can be considered. This cumulative approach necessitates an adjustment for multiplicity that makes the procedure conservative. However, this procedure can still be helpful in detecting a treatment effect at a population level with a certain power.

## Conflicts of Interest

The authors declare no conflicts of interest.

## Data Availability

Data sharing is not applicable to this article as no datasets were generated or analyzed during the current study.
